# Pulpotomy for the Management of Irreversible Pulpitis in Mature Teeth

**DOI:** 10.7759/cureus.51837

**Published:** 2024-01-08

**Authors:** Utkarsh Umre, Shweta Sedani, Aditya Patel, Akansha Bansod, Simran Kriplani

**Affiliations:** 1 Conservative Dentistry and Endodontics, Sharad Pawar Dental College and Hospital, Datta Meghe Institute of Higher Education and Research, Wardha, IND; 2 Prosthodontics, Sharad Pawar Dental College and Hospital, Datta Meghe Institute of Higher Education and Research, Wardha, IND

**Keywords:** calcium hydroxide, apical periodontitis, irreversible pulpitis, mineral trioxide aggregate (mta), pulpotomy

## Abstract

Strict protocols for evaluating the pulp's preoperative state should be developed, along with a new classification scheme for the different pulp states, as case selection plays a major role in the effectiveness of adult pulpotomy. In this case report, a male patient, age 15, who had a carious lower left first molar underwent pulpotomy. The pulp's initial state was ascertained by pulse oximetry, electric pulp testing (EPT), and cold testing. The final diagnosis was symptomatic irreversible pulpitis. A 12-month follow-up period following the placement of mineral trioxide aggregate (MTA) (MTA Angelus Angelus, Londrina, Brazil; Clinician’s Choice, New Milford, CT) and tooth-colored composite restoration revealed no visible anomalies in the postoperative radiographs, and the tooth remained functional and free of symptoms.

## Introduction

When managing pulpitis in mature permanent teeth, an adult pulpotomy is a biologically sound alternative to total pulpectomy in endodontics. By preserving the salvageable tooth structure, this technique lowers the risk of tooth fracture and maintains the vitality and defensive function of the residual pulp tissue. Adult pulpotomies for teeth with irreversible pulpitis have been investigated in several prior trials, with success rates varying between 78.1% and 98.4% [1]. The success of an adult pulpotomy can be significantly influenced by the pulp's preoperative condition. Clinicians frequently use sensibility testing, especially electric pulp testers, to evaluate the sensitivity of teeth, rather than relying solely on patient feedback and their interpretation. Furthermore, even after the pulp has degenerated, the nerve fibers might still be functional. Pulse oximetry and laser Doppler flowmetry are two newer technologies that can be used instead of pulp susceptibility testing to assess the health of pulp more accurately [2-5].

Regarding its impact and contact with the left-over pulp tissue, the capping substance may have an impact on the success of adult pulpotomy. The treatment of vital pulp with calcium hydroxide (CH) was common before the implementation of mineral trioxide aggregate (MTA), which later became the restorative material. One of the earliest histological investigation comparisons of CH and MTA as a pulp capping material on human permanent molars was published [6-9]. According to the study, thicker bridges were produced in all MTA samples, an odontoblastic layer was frequently present, and hyperemia was uncommon. Contrarily, CH cannot seal, has a tendency to resorb over some time, and does not attach to dentine, and dentinal tubules can serve as conduits for microleakage. The findings also demonstrated that in CH trials, necrosis was more common and more severe, there was no odontoblastic layer, and inflammation was more common. Eghbal et al. have done a histological evaluation of the favorable outcome of pulpotomy using MTA as a restorative material in mature molar teeth with irreversible pulpal inflammation that were scheduled for extractions [10]. According to the research conducted, each sample needed to be completely bridged with the pulp in the radicular area and was devoid of any bacterial products. In this case report, a mature carious molar with irreversible pulpitis underwent a coronal pulpotomy procedure with the MTA capping technique.

## Case presentation

**Figure 1:**
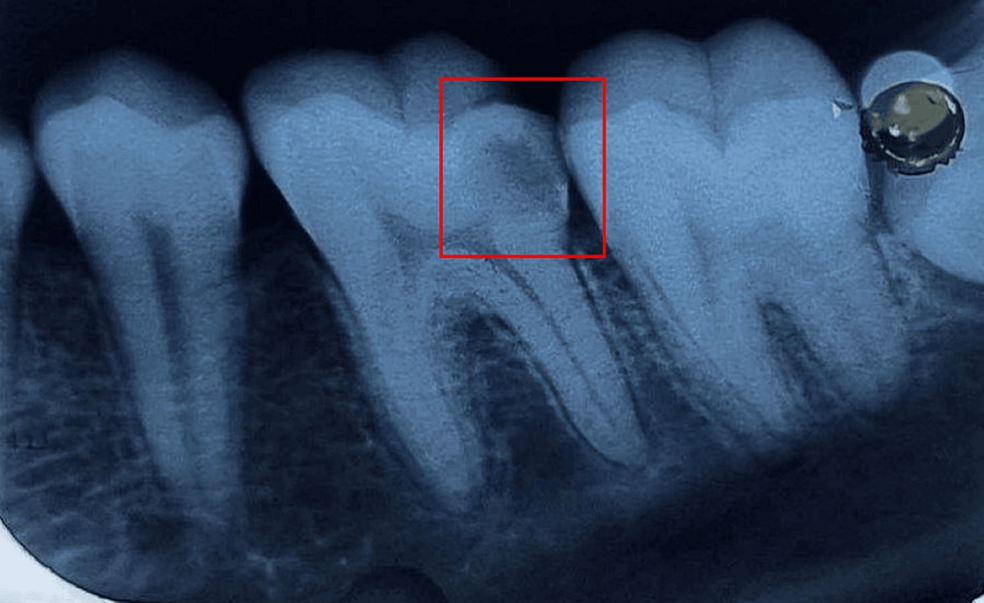
Radiographic evaluation of lower left first molar suggestive of irreversible pulpitis Radiographic evaluation of the lower left first molar shows radiolucency extending up to the pulp chamber on the distal pulp horn and sharpshooting pain on mastication, suggesting symptomatic irreversible pulpitis.

**Figure 2:**
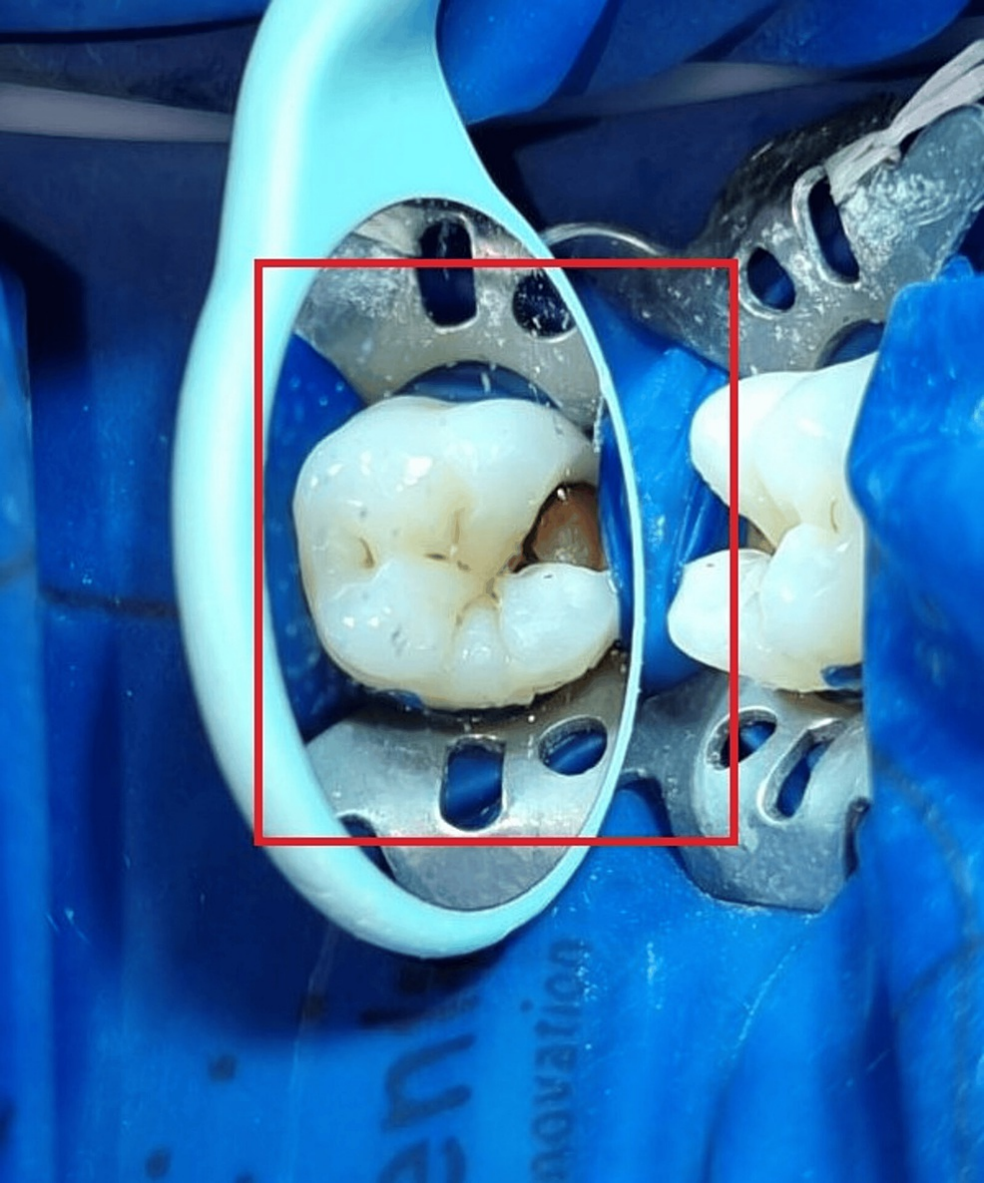
Pre-operative image

The Sharad Pawar Dental College and Hospital's outpatient department referred a male patient, age 15, who had a carious lower left first molar. The preliminary diagnosis following clinical and radiographic evaluation was symptomatic irreversible pulpitis of the lower left first molar destructed due to a carious lesion, as seen in the intraoral periapical radiograph in Figure 1. Figure 2 shows the preop image.

**Figure 3:**
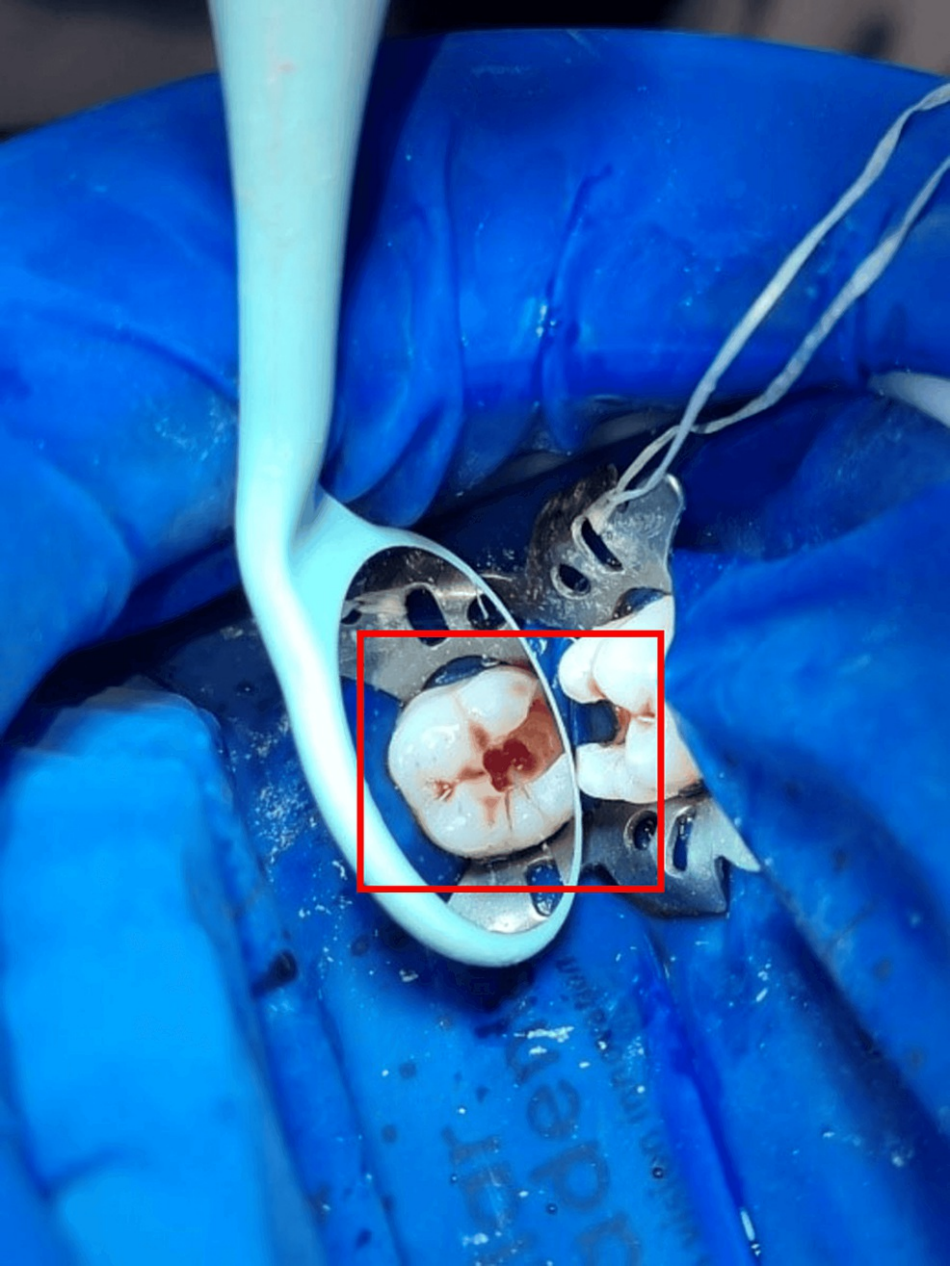
Removal of carious dentin and access cavity preparation

After primary assessment and recognition, patients were informed about the procedure and written consent was obtained following a thorough discussion of the follow-up period with a focus on the potential outcomes. Pulse oximetry, electric pulp testing, and cold testing were used to determine the pulp's initial state. The definitive diagnosis was symptomatic irreversible pulpitis. To provide local anesthesia, 1.8 mL of 2% mepivacaine hydrochloride was mixed with levonordefrin 1:20,000. Following rubber dam isolation and anesthesia, a carbide round bur was used to remove carious dentin. A size #3 carbide round bur was used to get access to the cavity, and an Endo-Z bur (Dentsply, USA) was used to de-roof the cavity and refine it. After deroofing and preparing the access cavity, pulp excavation was carried out to the level of the orifices using a sharp spoon excavator and a size #3 high-speed carbide round bur (Premier, USA) (Figure 3).

**Figure 4:**
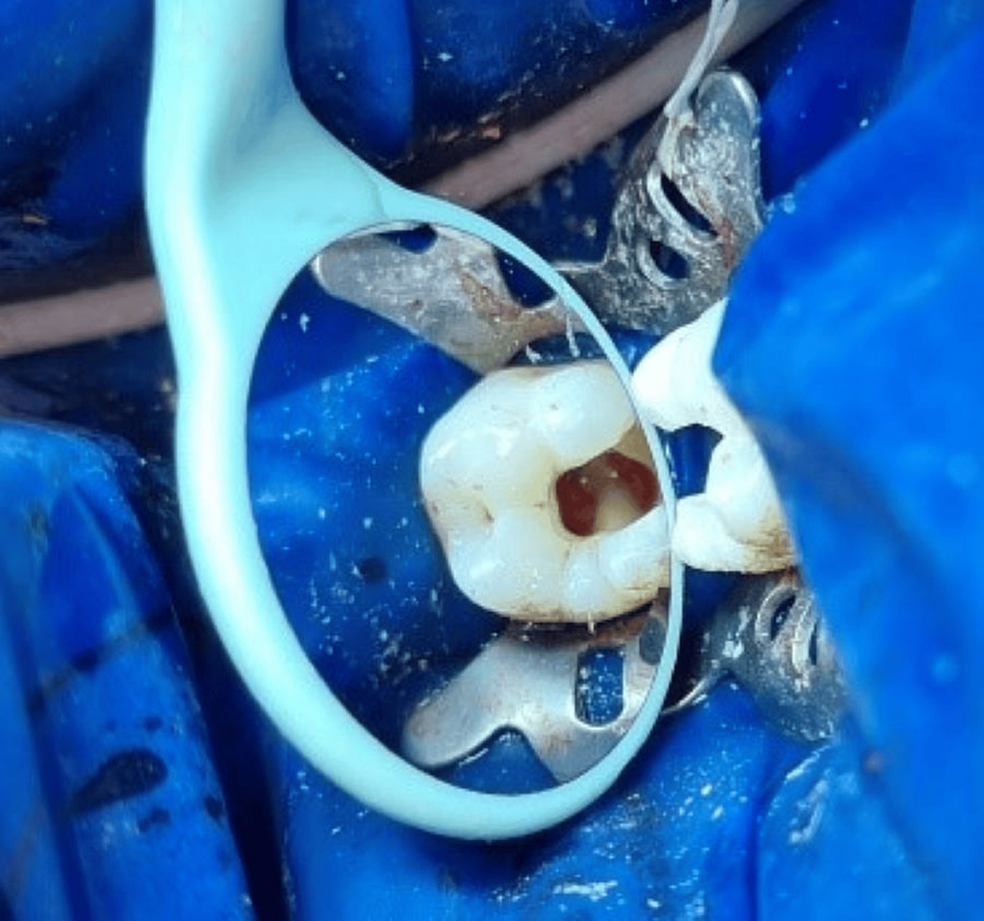
Hemostasis established using a cotton pellet soaked in NaOCl for two minutes

Sodium hypochlorite (NaOCl) was used to flush out the pulp chamber, and hemostasis was established by using a cotton pellet soaked in 5.25% NaOCl for two minutes (Figure 4).

**Figure 5:**
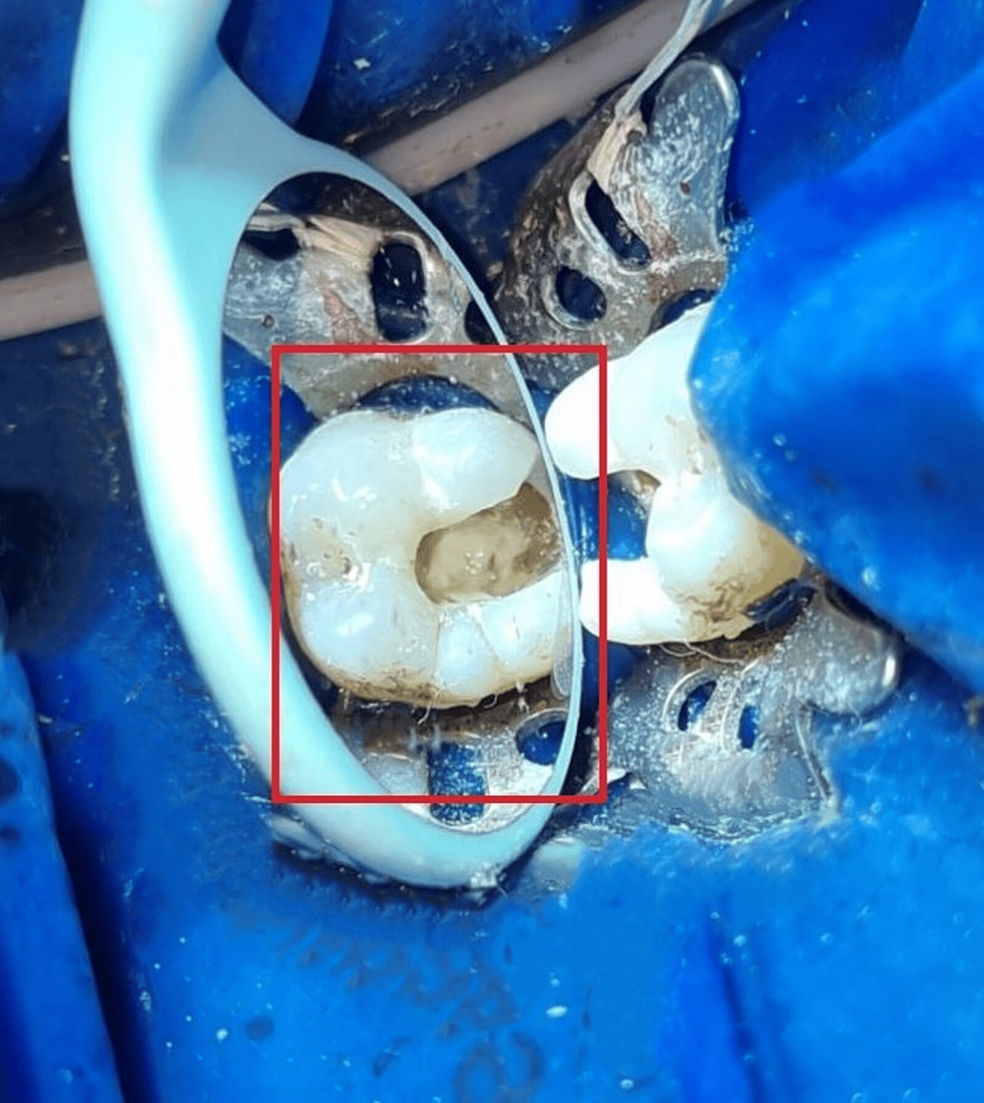
MTA placement done MTA - mineral trioxide aggregate

**Figure 6:**
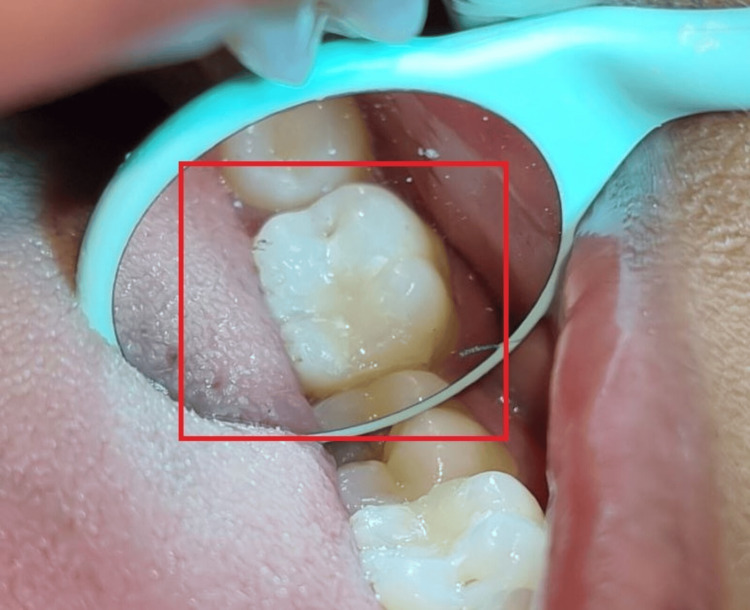
Composite restoration done after MTA placement

After adding a drop of sterile water to a dapen dish containing MTA powder (MTA, Angelus, Brazil), the powder and liquid were gradually mixed in a 3:1 ratio until the powder particles were completely wet. A wet cotton pellet was immediately applied on top of the MTA seen in Figure 5. After setting of MTA tooth-colored composite (3M, ESPE, USA) restoration was done as seen in Figure 6.

**Figure 7:**
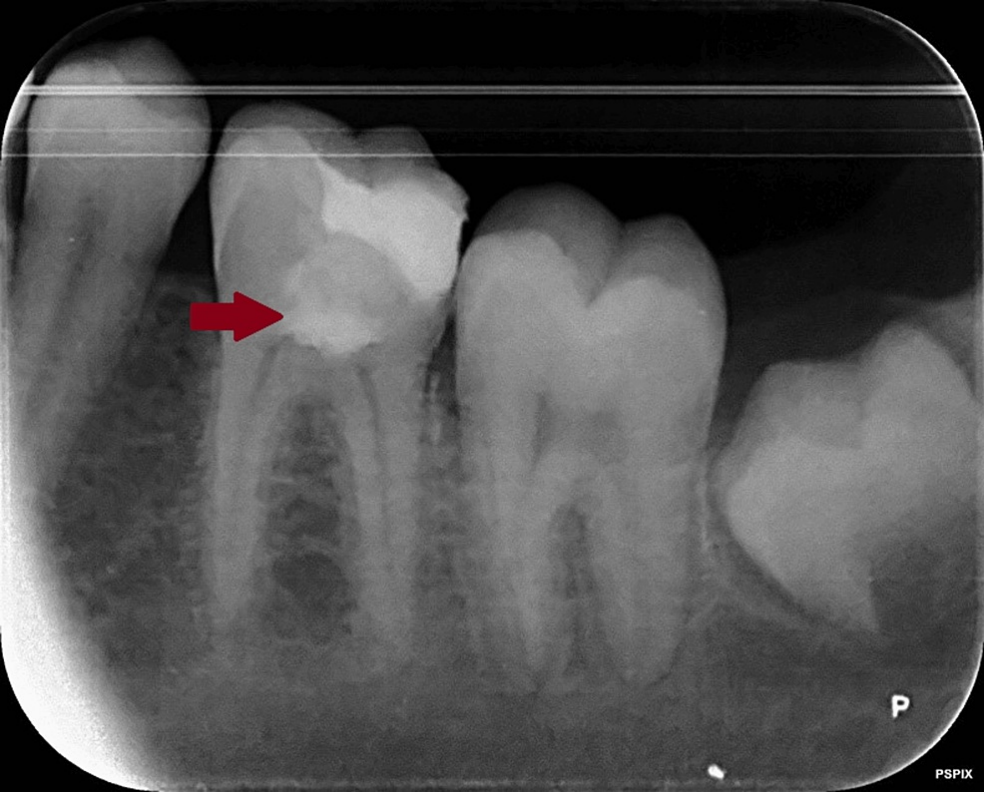
Twelve months follow-up

Both radiographically and clinically, the patient was assessed. The tooth was still functional and symptom-free after a 12-month follow-up period, and there were no obvious abnormalities (this could include no signs of infection, structural damage, or other concerns that might be visible in the x-rays) in the postoperative radiographs seen in Figure 7. Furthermore, clinical examination revealed no coronal discoloration.

## Discussion

Root canal therapy, which is a preventative procedure, is generally used to treat teeth with irreversible pulpitis since the pulp present in a root canal is frequently clear of bacteria and its products, and the idea is to render the root canal system sterile [11]. Considering the newer technologies and biomaterials, root canal treatments still present a challenge to physicians due to the intricacy of the pulp space and system and the related procedures to be undertaken [12]. The root canal procedure also renders a tooth inert by removing a significant percentage of tooth structure, which causes recurrent fractures of such teeth and eventual tooth loss. As a result, teeth like those without periapical lesions might undergo a pulpotomy, a vital pulp therapy method where the radicular pulp remains intact, and the coronal pulp is removed to keep the tooth vital. To prevent additional injury and to start the healing and repair process, an appropriate biocompatible material is put onto the remaining radicular pulp [13-15].

Given the progress made in the last two decades in the development of MTA-hydraulic calcium silicate-based materials, as well as our increased comprehension of pulp cytology, vascularization, and regeneration, coronal pulpotomy has been proposed as the sole treatment option for teeth that are mature [16-18]. By earlier investigations, mechanical pressure was used in this instance to establish hemostasis using a cotton pellet saturated in saline. In this instance, the bleeding time was two minutes, and hemostasis was attained in that time [19]. According to several studies, achieving hemostasis should not take longer than 10 minutes in pulpotomy instances for good management. MTA is a hydrophilic calcium silicate restorative material with good sealing, physical, and biological qualities that hardens in the surrounding moisture.

Regarding the final filling, the composite layer offered compressive and tensile strength as well as resistance to water sorption. The access opening was filled with conservation of the residual tooth structure, and the bonded composite restoration was effective during the follow-up period without a set crown over the tooth restored earlier [20,21].

According to Galani et al., the patient had a 12-month follow-up, and using the success and failure criteria, this case was considered successful. The success and failure criteria are likely predetermined guidelines or measures that help assess the outcome of a medical or dental intervention. These criteria could include various factors such as the absence of symptoms, functional status of the treated area, radiographic (x-ray) evidence, and possibly other clinical indicators. The authors may have defined specific parameters or conditions that, if met, would classify the case as successful, while the failure criteria would encompass conditions that indicate an unsuccessful outcome) [22]. Adult pulpotomy success depends heavily on case selection, so strict procedures for assessing the pulp's preoperative condition should be created, as should a new classification system for the various pulp states.

## Conclusions

The success of mature tooth pulpotomy in addressing irreversible pulpitis is evident in our patient outcomes, providing a compelling case for its consideration. Biomaterials like MTA have played a transformative role, challenging traditional endodontic treatment concepts by introducing pulp healing characteristics. The use of MTA reflects a paradigm shift toward materials that not only address immediate issues but also support the regeneration of pulp tissue. In the context of case selection, factors such as age, pulp condition, and bleeding control emerge as pivotal considerations. These variables underscore the personalized nature of endodontic care, where treatment choices are tailored to individual patient needs and the specific characteristics of the affected tooth. The dynamic interplay between evolving biomaterials and established protocols highlights the ongoing refinement of endodontic practices, enhancing the options available for effective and patient-centered dental care.
